# Revised Common Rule Changes to the Consent Process and Consent Form

**DOI:** 10.31486/toj.19.0055

**Published:** 2020

**Authors:** Leah L. LeCompte, Sylvia J. Young

**Affiliations:** ^1^ Department of Rehabilitation Services, Ochsner Clinic Foundation, New Orleans, LA; ^2^ Human Research Protection Program, Ochsner Clinic Foundation, New Orleans, LA

**Keywords:** *Consent forms*, *ethics committees–research*, *informed consent*

## Abstract

**Background:** The Federal Policy for the Protection of Human Subjects—the Common Rule—was revised in 2017 to reduce administrative burdens for low-risk research while enhancing protections for human subjects enrolled in greater-than-minimal-risk trials. These enhanced protections involve changes to the consent process.

**Methods:** We review the general requirements applicable to the consent process, as well as the additional elements of consent mandated by the revisions to the Common Rule. The regulations apply to federally funded studies and are optional for non–federally funded studies.

**Results:** Two new general requirements for the consent process, one basic required element for the consent form, and three optional additional elements for the consent form were added in an effort to improve potential subjects’ understanding of research studies and to facilitate the exchange of information between the research staff and potential subjects. Important information about the study should be extracted into a concise key information section to help potential subjects make informed decisions regarding participation.

**Conclusion:** The revisions to the Common Rule are intended to enhance human subject protection by providing more information in an understandable form during the consent process. The new consent elements aim to increase transparency and help improve clarity.

## INTRODUCTION

Respect for human dignity and individual autonomy is one of the basic principles of research ethics.^1,2^ Consequently, voluntary informed consent is required from potential subjects before they can be enrolled in a research study. Obtaining this consent involves ongoing interaction between the research staff and the potential subject. The consent process should ensure that subjects have the information they need to make an informed decision about whether or not to participate. In addition to verbal discussions, the written consent form plays an important role in the consent process and should be used as a guide for the exchange of information between the research staff and the potential subject. The consent form should not only facilitate discussions that expand the subject's understanding of the study, but it also serves as a reference for the potential subject to take home as he/she considers whether to enroll in the study.^3-8^

The Federal Policy for the Protection of Human Subjects, known as the Common Rule, is a set of regulations that guide the ethical conduct of human subjects research. Revisions to the Common Rule were published on January 19, 2017 with a general compliance date of January 19, 2019, the first significant changes to the rule since it was published in 1991. The revisions to the regulations changed the consent form to improve potential subjects’ understanding of a research study and to accommodate changes in the research environment. The regulations apply to federally funded studies and are optional for non–federally funded studies. The goal of the revisions is to promote subjects’ autonomy so that they can make informed decisions regarding participation in a research study.

We review the changes to the general requirements for the consent process and the new consent form elements mandated in the revised Common Rule.^8-14^

## TWO NEW GENERAL REQUIREMENTS

When a potential subject is invited to participate in a research study, the following general requirements apply to the consent process:

1. Informed consent of the subject or the subject's legally authorized representative must be obtained prior to involving the subject in research.
2. Investigators must give the potential subject or the legally authorized representative the opportunity to discuss and consider whether or not to participate, and the investigator must minimize coercion or undue influence.
3. Informed consent information must be in language understandable to the subject or the legally authorized representative.
4. Information must be provided that a reasonable person would want to know to make an informed decision about whether to participate, and there must be an opportunity to discuss that information.
5. Informed consent must begin with a concise and focused presentation of the key information that is organized and presented in a way to facilitate understanding of the reasons why one might or might not want to participate.
6. Informed consent cannot include exculpatory language through which the subject or legally authorized representative is made to waive any of the subject's legal rights or that releases the investigator from liability for negligence.^9^

Requirements 4 and 5 are new. The “reasonable person” standard is used to clarify the level of information that potential subjects would want to know before deciding if they’d like to participate. Potential subjects should have the opportunity to engage in ongoing discussions with the research staff to ensure understanding. The other new requirement is to begin the consent form with a brief and focused summary of the information that is most relevant to the decision-making process.^6^ The summary should include a statement that the subject's participation is voluntary, an explanation of the purpose of the research, and a description of the procedures, as well as how long the subject will be involved in the study, the risks, the benefits, and the alternatives if the subject decides not to participate. ^6,8,9-11,15-20^

The key information summary addresses a problem in the consent process. Research has documented that consent forms have become lengthy and complex, and many subjects therefore have limited understanding of the research for which they are providing consent.^3^ A 2009 review of the literature found that fewer than one-third of subjects adequately understood important aspects of their studies, such as study goals, risks/benefits, and randomization.^21^ Many consent documents are written at a reading level that is higher than the average recommended eighth-grade level, but almost half of American adults read at or below the eighth-grade level.^3,22^

The reasonable person standard for presenting information and the key information summary can help subjects with the decision-making process by facilitating discussion with the research staff and by presenting significant information in a transparent and easy-to-understand manner.

## FOUR NEW CONSENT FORM ELEMENTS

The revised Common Rule adds four new elements to the consent form: a required basic element (increasing the number of basic elements from 8 to 9) and three additional elements that should be included only if applicable (increasing the number of additional elements from 6 to 9). The new required basic element addresses the collection of identifiable private information or identifiable biospecimens, and the three new additional elements address commercial profit, return of clinically relevant research results to the subject, and whole genome sequencing. The Ochsner Clinic Foundation Research Informed Consent template that incorporates these changes is included as an addendum to this article. A summary of the four new consent form elements is provided in the Table.

**Table.** Summary of New Consent Form Additional Elements

When the research involves	Include in the consent form
the collection of identifiable information or identifiable biospecimens	a statement indicating whether identifiers may be removed and if the deidentified information or biospecimens may or may not be used or shared for future research
use of biospecimens	a statement indicating whether biospecimens may be used for commercial profit and if the subject will share in that profit
clinically relevant results	a statement indicating whether clinical results, including individual research results, will be returned to the subject and if so, under what conditions
whole genome sequencing	a statement indicating that the research will/will not/might involve whole genome sequencing

### Consent Form Required Basic Elements

All basic elements of consent must be included in the consent form.^9^ The revisions to the Common Rule did not change the original eight basic elements of consent:

1. Explanation of the purpose, duration of participation, and procedures
2. Risks
3. Benefits
4. Alternative procedures
5. Information about confidentiality, compensation, and treatments if injury occurs
6. Compensation or medical treatment if injury occurs in studies greater than minimal risk
7. Contact information
8. Statement that participation is voluntary; no penalty or loss of benefit if subject refuses to participate or if they withdraw from the study^9^

The new basic element requirement is to include one of the following statements if the research involves the collection of identifiable private information or identifiable biospecimens:

- The identifiable private information or identifiable biospecimens may be used for future research or distributed to other researchers without additional consent after identifiers have been removed.

OR

- Private information or biospecimens will NOT be used for future research even if identifiers are removed.^9,11^

Deidentification involves removing information about subjects so that information or biospecimens are not likely to be linked back to them (ie, name, address, phone numbers, email address, Social Security number, photos, fingerprints). Historically, subjects have not been informed that their deidentified private information and/or deidentified biologic specimens (eg, blood, tissue samples, urine) may be used or shared with other researchers for additional research without their knowledge.^12^ One well-known example is the use of cancer cells removed from Henrietta Lacks without her knowledge to establish a cell line that was reproduced and distributed for a multitude of research studies.^16,23^ In 2004, the Havasupai Tribe sued the Arizona Board of Regents and the Arizona State University researchers for using DNA samples that were collected for studies on type 2 diabetes but were later used in other studies to research mental illness and theories of the tribe's geographic origins that contradicted their traditional stories.^23^ Clarifying the private information/biospecimen issue will decrease concerns related to unauthorized sharing of data and biospecimens.^8^ Subjects have the right to know that their deidentified information or specimens may be used for future research without their additional consent.^15^

Studies have shown that subjects are generally willing to consent to the use of their biospecimens in research; however, subjects want to be asked for their consent.^24^ Not having the opportunity to give their permission for this use often leads to lack of trust and feeling deceived.^25,26^ One study showed that 90% of subjects felt it was important for researchers to ask their permission.^27^ Education should be provided to subjects on how their information and/or biospecimens will be stored and the potential contribution to other research studies. This education should include a conversation about individual risks and societal benefits. D’Abramo et al reported that subjects often overestimate the individual benefits, which are nonexistent, and are unaware of risks^28^ that can include insurance discrimination, employment issues, psychological harm, and family disruption. In addition, researchers should not guarantee confidentiality.^24^ Once education is provided during the consent process, subjects are given the opportunity to make their own decisions regarding participation.^29^

The Ochsner informed consent template language for this new required basic element is as follows:

We may use or share your research information and/or biospecimen for future research studies, but it will be deidentified, which means that it will not contain your name or other information that can directly identify you. This research may be similar to this study or completely different. We will not ask for your additional informed consent for these studies. We may also share your deidentified information and/or biospecimen with other researchers at Ochsner or at other institutions.

Other examples of personal information/biospecimen text are as follows:

- Any personal information that could identify you will be removed from your sample. Your sample may be used for future research studies without the investigator asking for your additional permission.^30^
- Any personal information that could identify you will be removed from your sample. Your sample will not be used for any future research studies.^30^
- Your name and other information that could identify you will never be released into a scientific database.^31^

The purpose of adding this new basic element requirement is to inform subjects of what might happen in the interest of full disclosure. This new basic element does not require that subjects be given a choice to opt out of having their deidentified data or biospecimens used for future research. If subjects do not wish to allow their deidentified data or biospecimens to be used or distributed, their only choice may be to not participate in the research.^16^

### Consent Form Additional Elements

The additional elements of consent are statements that must be included in the consent form if the information is relevant to the research. The revisions to the Common Rule did not change the original six additional elements of consent:

1. Statement about potential risks to the subject or to the embryo or fetus if the subject is or becomes pregnant
2. Circumstances under which subject's participation may be terminated
3. Any additional costs
4. Consequences of a subject's decision to withdraw from the research and procedures for termination of participation
5. Statement that significant new findings that may relate to the subject's willingness to continue participation will be provided
6. Approximate number of subjects involved^9^

The revision to the Common Rule mandates three additional elements that must be included in the consent form if they are relevant to the research:

1. A statement indicating whether biospecimens may be used for commercial profit and whether or not the subject will share in that profit
2. A statement indicating whether the results will be given to the subject and if so, under what conditions
3. A statement indicating that the research will, will not, or might include whole genome sequencing^9^

#### Commercial Profit

Many subjects are not aware that their tissue has any commercial value.^32^ Subjects must be informed of the possibility that research using their biospecimens could lead to the development of commercial products, and whether the subjects will share in any commercial profits must be made clear.^24^ Courts have held that individuals do not hold property rights to their donated tissue and cells. One example is *Moore v Regents of the University of California*. Moore was being treated for a rare form of cancer and signed a surgical consent form for removal of his spleen. Prior to the surgery, his physician was aware that the cells from Moore's spleen could have commercial value. A cell line was developed from Moore's tissue that helped produce drugs which may be beneficial in the treatment of leukemia and possibly for acquired immunodeficiency syndrome. The cells were valued at approximately $3 billion.^33^ Moore sued for damages based on the failure of his physician to inform him of the commercial gains made from his cells but lost when the case was referred to the California Supreme Court.^33^ The requirement that investigators disclose the potential for commercial profit and whether subjects will share in that profit is intended to enhance the subjects’ understanding because such knowledge may affect their willingness to participate in a study.^11,12^

The Ochsner informed consent template provides an example of how the new additional element regarding commercial profit can be written:

Data or biospecimens collected from you for this research may be used to develop new tests, drugs, or devices. Your samples may be used for commercial profit and there is no plan to share these profits with you.

Another example of the commercial profit language is “Your sample may be used to develop new drugs or other products for commercial purposes. If these products make money, there are no plans to share the money with you.”^30^

#### Return of Results

Consent forms must now include a statement regarding whether clinically relevant research results, including individual research results, will be disclosed to subjects, and if so, under what conditions.^11^ Disclosure of results in research has been the subject of much debate.^34,35^ Some authors argue that, based on the principle of respect for persons, disclosure should be routine in research, while others argue that respect for persons is not violated when results are not returned.^36,37^ Some authors think results should be made available only to subjects who desire the results and that subjects should have the option of receiving all their research results or just those with clinical significance.^38^ Others believe only clinically relevant research results should be returned because the information can have a positive impact on the subject's health.^39^ Beskow and Burke recommend returning results when the probability of a serious condition that has an effective intervention is high.^34^ An example includes a mutation known to result in high risk for colon cancer; having this information would allow subjects to pursue aggressive screening.^25^ Others believe that results should not be returned because returning results can cause psychological risks (anxiety about the discovery of future disease or uncertain information); stress for family members; social risks of stigmatization or discrimination; and issues with confidentiality, insurability, and employment.^24,38,40-43^

In contrast, research has documented that 96% of subjects have a strong preference for receiving results.^44^ Subjects express a feeling of ownership of the findings. Some subjects believe returning results is a moral obligation of the investigator. Other subjects feel that learning results is a benefit of their participation. Some subjects consider results as an acknowledgment of their contribution to research, a sign of respect. However, although subjects want to know the results of the studies they have participated in, results are rarely returned.^43-47^

Research differs from treatment. The goal of research is to produce generalizable knowledge for future patients, whereas in the clinical setting, the goal is to provide the best possible treatment for the patient. Some argue that returning individual results may promote therapeutic misconception.^43^ Therapeutic misconception occurs when subjects believe that the purpose of the research is to advance their own interests by providing them with medical benefits rather than to advance human knowledge.^3,33,36,43,48,49^

As these examples demonstrate, the question of disclosing research results is controversial. Attention is needed during the consent process to clarify whether results will be returned to subjects and under what conditions.^25,41,49^ The consent form should specifically state whether or not results will be returned.^36^ Subjects should not enroll in a study thinking they will get information about their health or the results of the study if they will not.

The Ochsner informed consent template provides examples of how the new additional element regarding returning of results can be written:

We may learn things about your health as part of this research that may affect your treatment. If this happens, this information will be provided to you. You may need to meet with professionals with expertise to help you learn more about your research results. You can discuss this information with your doctor.

OR

We may learn things about your health as part of the research; however, we will not share this information with you because…

Other examples of the returning results language follow:

- Results of research testing on your sample will be returned to you.^30^
- Results of research testing on your sample may be given to you or your doctor. This will be done only if the results may be necessary for your care.^30^
- You should not expect to get individual results from research done using your sample.^31^
- We will offer to tell you a finding like this only if it is about a disease that is likely to cause early death if not treated.^31^

#### Whole Genome Sequencing

Subjects must be informed if the research will, will not, or might include whole genome sequencing. Whole genome sequencing is increasingly being used in research to identify genetic variations and is expected to continue to expand. Whole genome sequencing provides information that could predict subjects’ future medical conditions and has the potential to impact an individual's family members.^50,51^ Ethical and practical concerns related to whole genome sequencing include storage, analysis, and return of results. Because every person's DNA is unique, the realization that fully guaranteeing privacy may be impossible is becoming increasingly evident.^52-54^

Communicating the concept of whole genome sequencing to subjects can be challenging, and whether subjects gain sufficient understanding of all the implications of whole genome sequencing is unclear. In one study, more than 40% of subjects reported that they did not know they were enrolled in a genetic research study, 53% did not know if they had already given DNA to their doctors, and 62% did not know if their DNA would be stored as part of a genetic research study.^55^ The informed consent process should help subjects understand the implications of whole genome sequencing, the possible results, and the limitations of testing to help them make decisions about participation.^12,23^

The potential uses and outcomes of whole genome sequencing may currently be too extensive to know, explain, or understand.^22^ Albert Einstein may have summed it up best when he said: “If you can’t explain it simply, you don’t understand it well enough.”^56^ The need for more effective ways to communicate information about whole genome sequencing during the informed consent process to subjects is growing. Genetic counselors are often involved.^7,57^ Providing verbal explanations along with written materials has been suggested to improve subjects’ ability to comprehend the information.^21,52^

The Ochsner informed consent template provides an example of how the new additional element regarding whole genome sequencing can be written to facilitate an ongoing discussion between the research staff and a potential subject:

This research will/will not/might involve whole genome sequencing. Please ask the principal investigator or study team if you have any questions about how your genetic information will be used.

Another example of the whole genome sequencing language is “Research testing on your sample will include whole genome sequencing. This means we will map your entire genetic code. If you have questions about this ask the study staff.”^30^

## CONCLUSION

The revisions to the Common Rule are intended to enhance human subjects protection by providing more information during the informed consent process than was provided under the prior regulations. The intent of the revisions is to make consent forms clear and focused on providing information up front so that potential subjects can make informed decisions. The new consent elements aim to increase transparency and help improve clarity. These changes call for providing information that is relevant to a subject's decision-making process and that promotes autonomy. Consent forms should facilitate discussions to ensure understanding. Education of institutional review board members, investigators, and research organizations/institutions is needed to implement these changes. Making sure subjects have clear information that is easy to read and understand and that highlights the information most important to them when making decisions is an ongoing opportunity and challenge.

## Figures and Tables

**Figure:**
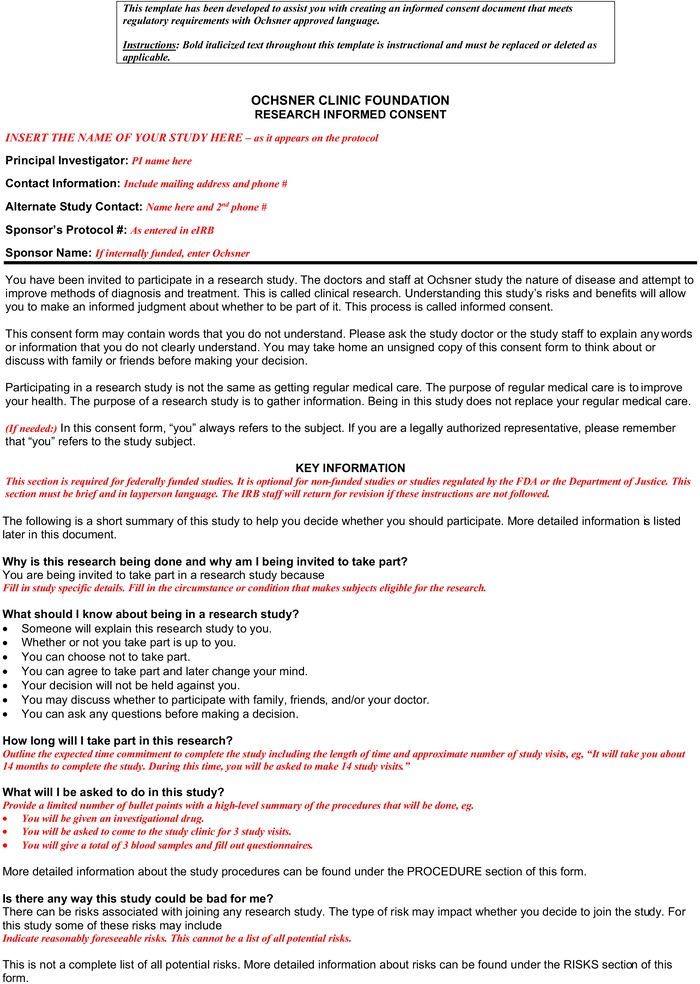


**Figure:**
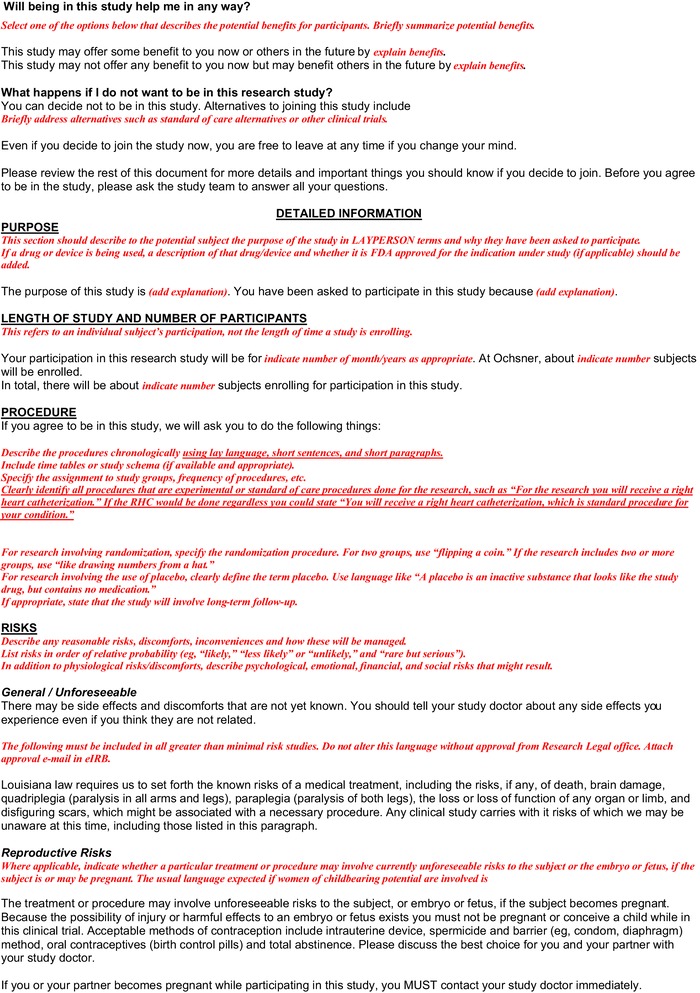


**Figure:**
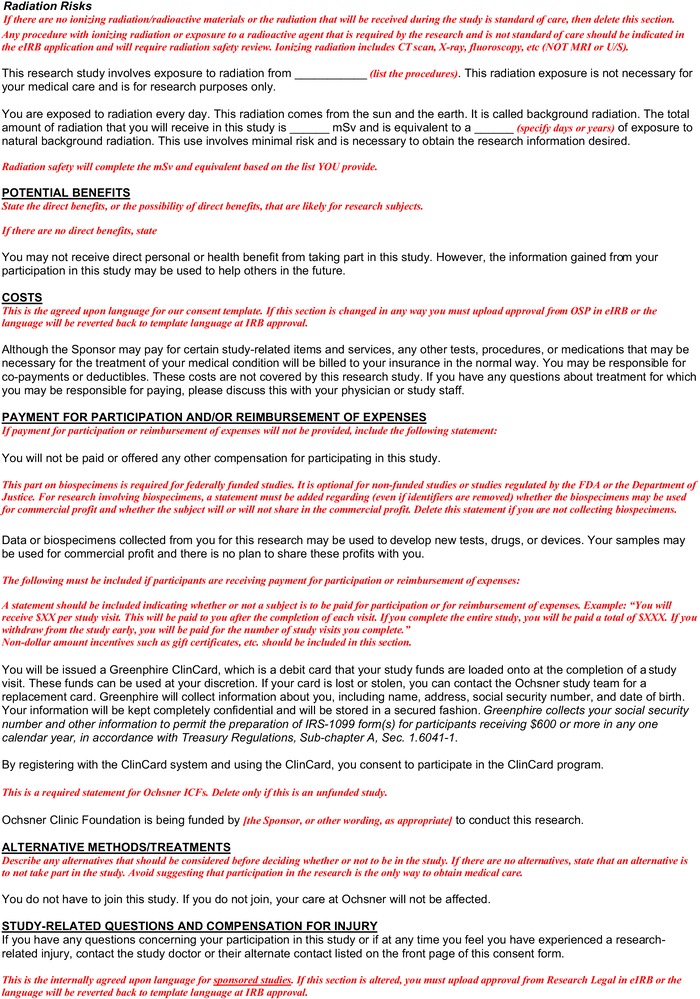


**Figure:**
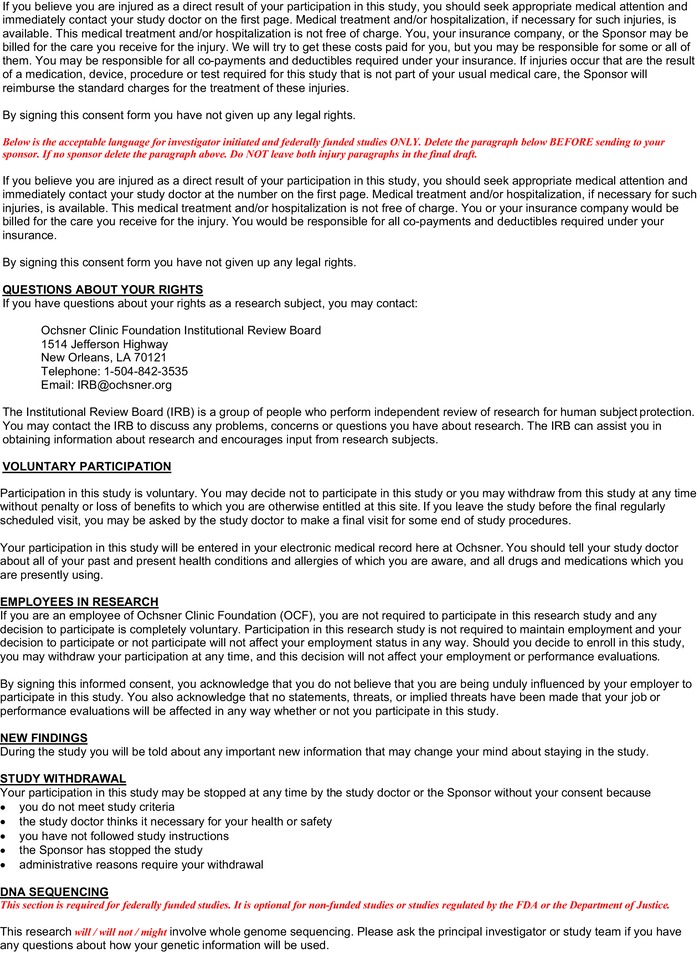


**Figure:**
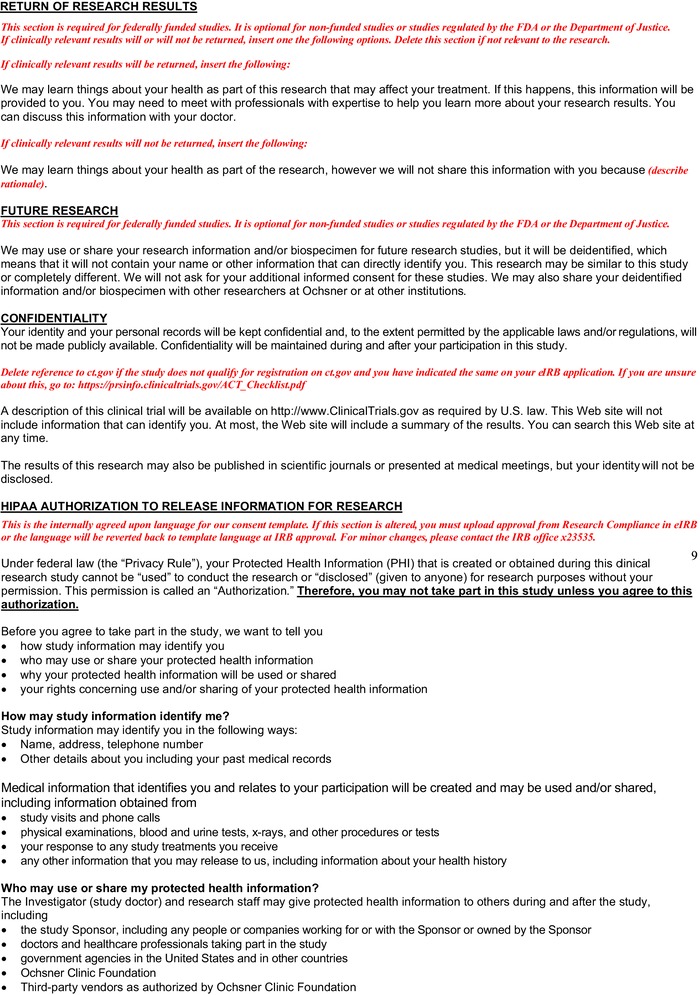


**Figure:**
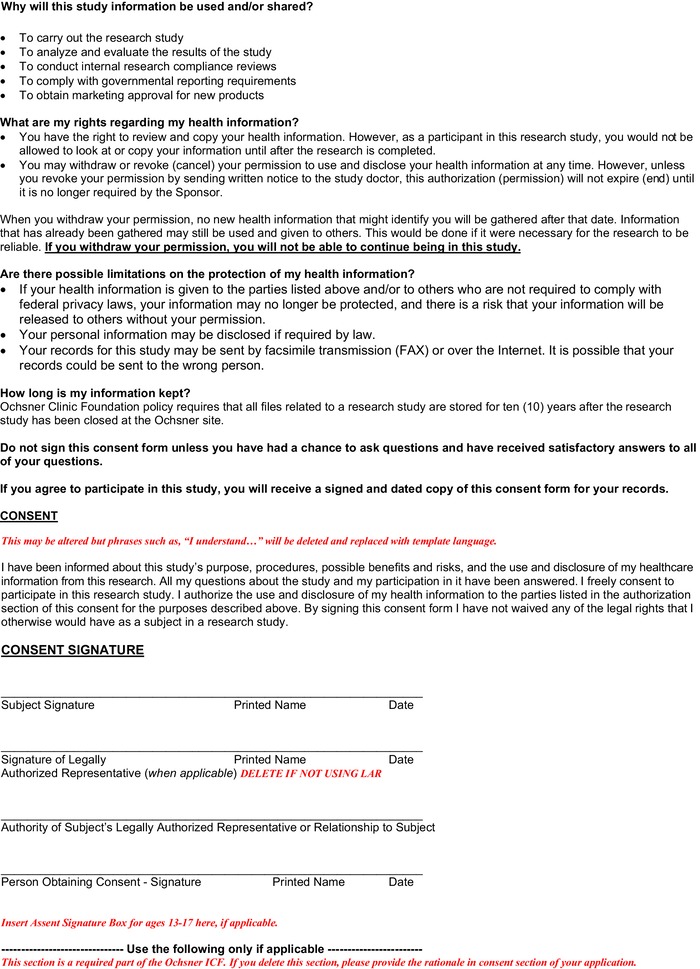


**Figure:**
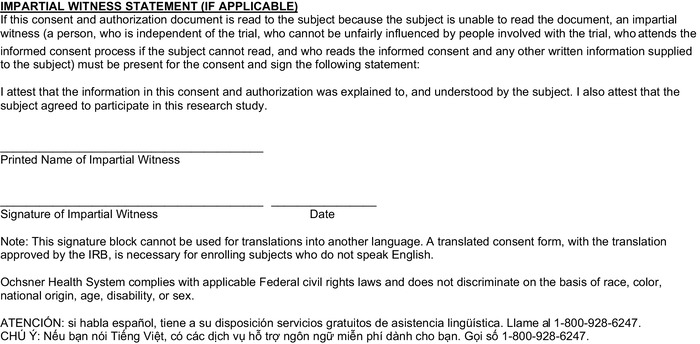


## Notes

This article meets the Accreditation Council for Graduate Medical Education and the American Board of Medical Specialties Maintenance of Certification competencies for Patient Care, Medical Knowledge, Interpersonal and Communication Skills, and Systems-Based Practice.
